# Adult-Onset Limb-Girdle Phenotype Associated With a Homozygous PYROXD1 Variant: The Case of a North African Family

**DOI:** 10.7759/cureus.117414

**Published:** 2026-10-04

**Authors:** Abraham Alhatou, Omar M Alhatou, Mohammed Alhatou

**Affiliations:** 1 DeBusk College of Osteopathic Medicine, Lincoln Memorial University, Knoxville, USA; 2 Medicine, Royal College of Surgeons in Ireland - Bahrain, Busaiteen, BHR; 3 Neurosciences, Hamad Medical Corporation, Doha, QAT

**Keywords:** adult-onset myopathy, clinical exome sequencing, congenital myopathy, genotype-phenotype correlation, joint hypermobility, limb-girdle muscular dystrophy, myofibrillar myopathy, pyroxd1

## Abstract

Mutations in the pyridine nucleotide-disulfide oxide reductase domain-containing protein 1 (PYROXD1) gene are characteristically linked to early-onset congenital myofibrillar myopathy, typically presenting with generalized weakness, hypotonia, and respiratory insufficiency in infancy or early childhood. However, emerging evidence suggests phenotypic heterogeneity.

We report the case of a 45-year-old Tunisian woman, born to non-consanguineous parents, who presented with a slowly progressive limb-girdle muscular dystrophy (LGMD) phenotype; her older brother, subsequently confirmed to carry the same homozygous variant, had a similar but more severe clinical history of proximal weakness. The patient’s symptoms began at age 17 with running difficulties, progressing by age 35 to difficulty rising from a seated position and climbing stairs, and by age 45 to exertional dyspnea, dysphagia, and sleep-disordered breathing. Physical examination revealed a high-arched palate, scoliosis, joint hypermobility, limited ankle dorsiflexion, palatal weakness, and mild neck flexion weakness alongside a proximal-dominant limb-girdle pattern of weakness. Her brother showed a matching but more severe phenotype starting at age 12. Electrodiagnostic studies demonstrated normal nerve conduction and a non-irritable myopathy; serum creatine kinase was entirely normal, and pulmonary function testing revealed a restrictive ventilatory defect with mild obstructive sleep apnea on polysomnography. Clinical exome sequencing confirmed a pathogenic homozygous c.464A>G (p.Asn155Ser) mutation in the PYROXD1 gene in both affected siblings, with both parents confirmed as unaffected heterozygous carriers. This represents the first molecularly confirmed family with PYROXD1-related myopathy in Tunisia and North Africa, and only the second family identified across the African continent; a single unrelated Tunisian case harboring the same variant has also recently been reported.

This case expands the known clinical spectrum of PYROXD1 variants to include adult-onset LGMD-like presentations with respiratory and bulbar involvement, emphasizes the diagnostic utility of recognizing associated dysmorphic features (scoliosis, high-arched palate, nasal speech, and joint hypermobility), and highlights the pivotal role of comprehensive genomic testing in atypical adult myopathies.

## Introduction

Pyridine nucleotide-disulfide oxide reductase domain-containing protein 1 (PYROXD1) is a nuclear-localized oxide reductase essential for managing cellular altered redox environments and maintaining myofibrillar integrity. Biallelic (autosomal-recessive) pathogenic variants in PYROXD1 cause a rare, distinct congenital myopathy with myofibrillar pathological features, which should be distinguished from the classic myofibrillar myopathies, a genetically heterogeneous group most often caused by autosomal-dominant variants in genes such as DES, MYOT, LDB3/ZASP, FLNC, BAG3, and CRYAB. Affected infants with PYROXD1-related myopathy typically display generalized hypotonia, global motor delays, feeding difficulties, and respiratory insufficiency early in life, with muscle biopsies demonstrating hallmark myofibrillar disorganization, inclusions, and minicores [1,2].

As genetic testing has become more widely utilized in neuromuscular clinics, the phenotypic boundaries of many genetic myopathies are being redefined. Emerging literature indicates that PYROXD1 mutations can occasionally manifest as later-onset, milder, or restricted limb-girdle muscular dystrophy (LGMD) phenotypes, challenging the classical paradigm of this disease [3,4]. Unlike the classic congenital phenotype, this adult-onset presentation often lacks early hypotonia or infantile respiratory failure, and because serum creatine kinase (CK) and standard electrodiagnostic testing are frequently normal or only mildly abnormal, diagnosis can be substantially delayed unless clinicians maintain a high index of suspicion based on associated dysmorphic and connective-tissue features.

Despite these advancements, reports of PYROXD1-associated myopathies remain exceedingly rare worldwide, particularly within African populations. To date, only one family has been reported on the entire African continent [5], and a single unrelated adult-onset case has recently been reported from Tunisia [6]. Respiratory involvement, including restrictive ventilatory impairment, has also been increasingly recognized as a feature of PYROXD1-related myopathy, even in adult-onset, non-congenital presentations [2,3]. Here, we present the clinical, electrophysiological, and genetic findings of a 45-year-old female and her older brother, in whom targeted segregation testing confirmed the same homozygous PYROXD1 variant, with both parents confirmed as heterozygous carriers. This represents the first molecularly documented family in Tunisia and North Africa presenting with an adult-onset LGMD-like phenotype secondary to a homozygous c.464A>G (p.Asn155Ser) variant in PYROXD1.

This case was previously presented in preliminary form as a meeting abstract at the 2021 American Academy of Neurology Annual Meeting; we present the full clinical, electrophysiological, and genetic details for the first time here.

## Case presentation

A 45-year-old woman, born to non-consanguineous Tunisian parents, was referred to our neurology clinic for evaluation of a slowly progressive weakness in her proximal lower extremities. She had an unremarkable early childhood and no relevant past medical history. However, at age 17, she began noticing functional limitations, specifically experiencing difficulties running long distances and participating in high school gymnastics.

Over the subsequent two decades, her muscle weakness progressed in a slow, indolent manner. By age 35, her mobility was further compromised, and she developed significant difficulty rising from a seated position as well as ascending and descending stairs, requiring her to rely heavily on handrails for stability. By age 45, she additionally reported exertional dyspnea and became noticeably winded when attempting to verbalize long sentences. Around this time, her spouse reported new-onset snoring. The patient also manifested dysphagia with occasional choking on liquids. Interestingly, her older brother experienced a strikingly similar but more accelerated presentation, with proximal weakness beginning at age 12 and resulting in more severe overall functional impairment. Given the symmetric proximal pattern of weakness in both siblings, they were both initially diagnosed clinically with LGMD.

On physical examination, the patient demonstrated distinct dysmorphic and skeletal features, including a high-arched palate, moderate scoliosis, and generalized joint hypermobility. Her ankle dorsiflexion was restricted to 90 degrees bilaterally. A cranial nerve assessment was unremarkable except for noticeable palatal weakness and an associated nasal quality to her speech; there was no evidence of ptosis, ophthalmoplegia, or facial muscle weakness.

On manual muscle testing, mild weakness was noted in the neck flexors (scored 4+/5 or 5-/5). Upper extremity testing revealed mild proximal weakness involving the deltoid and biceps (5-/5) and more pronounced weakness in the triceps (4/5), while distal upper extremity strength was fully preserved. Lower extremity testing demonstrated clear proximal weakness, with hip flexion graded at 4/5, knee extension and foot dorsiflexion graded at 5-/5, and normal foot plantar flexion. Sensory examination was intact across all modalities. Deep tendon reflexes were diminished, yielding 1+ responses in the upper extremities and only trace, sluggish responses in the lower extremities. Formal standardized functional outcome measures were not administered as part of this initial evaluation; this is addressed further as a limitation below.

The patient presented with a slowly progressive, non-fluctuating phenotype without ptosis or ophthalmoplegia, arguing against a neuromuscular junction disorder such as myasthenia gravis. Although normal creatine kinase and electrodiagnostic testing (nerve conduction studies/electromyography, detailed below) supported a primary myopathic process without establishing a molecular diagnosis, the indolent disease course combined with a positive family history strongly favored an inherited myopathy, including a congenital myopathy. Given this familial clustering and the unique constellation of myopathy combined with skeletal abnormalities, we elected to pursue clinical exome sequencing directly as the primary diagnostic tool, rather than an invasive muscle biopsy or a non-specific muscle MRI. A genetic-first strategy is particularly efficient in cases of suspected inherited myopathy, maximizing diagnostic yield while minimizing unnecessary patient morbidity.

Initial laboratory workup, including creatine kinase, aldolase, thyroid function (thyroid-stimulating hormone: 1.86 mIU/L; free thyroxine: 19.8 pmol/L), lactate, and a metabolic profile, was unremarkable (creatine kinase 113 IU/mL; reference range <192 IU/mL). Autoantibody screening for connective tissue disease, specifically rheumatoid factor, antinuclear antibodies, and antineutrophil cytoplasmic antibodies, returned negative. Magnetic resonance imaging (MRI) of the lumbar spine, obtained using sagittal T1-weighted, T2-weighted, and short tau inversion recovery sequences and axial T1-weighted and T2-weighted sequences, was unremarkable, revealing no compressive radiculopathy or structural spinal cord lesions to account for her lower extremity weakness.

Pulmonary function testing demonstrated a restrictive ventilatory defect, evidenced by a reduced forced vital capacity of 2.07 L (63% of predicted) and a severely diminished peak inspiratory flow of 169 L/min (46% of predicted), juxtaposed against a relatively preserved peak expiratory flow of 380 L/min (88% of predicted). Polysomnography confirmed mild obstructive sleep apnea.

Electrodiagnostic evaluation was subsequently performed. Nerve conduction studies of the upper and lower extremities were entirely within normal limits, ruling out an underlying peripheral neuropathy or neuronopathy, as detailed in Table 1 and illustrated by representative tracings in Figure 1. Needle electromyography revealed a non-irritable myopathic pattern characterized by short-duration, low-amplitude motor unit action potentials (MUAPs) in the proximal musculature, without active denervation potentials such as fibrillations or positive sharp waves, as summarized in Table 2.

**Table 1:** Nerve conduction study

Nerve	Site	Latency (ms)	Amplitude	NCV (m/s)
Motor Nerve Conduction Study				
Median (L)	Wrist	3.0	10.8 mV	-
Median (L)	Elbow	6.6	9.8 mV	55.9
Ulnar (L)	Wrist	2.7	8.2 mV	-
Ulnar (L)	Distal Elbow	5.7	9.1 mV	62.1
Ulnar (L)	Proximal Elbow	7.3	8.6 mV	64.1
Peroneal (R)	Ankle	3.9	1.9 mV	-
Peroneal (R)	Head of Fibula	10.9	1.5 mV	47.1
Peroneal (R)	Popliteal	12.4	1.4 mV	51.6
Tibial (R)	Ankle	4.3	11.9 mV	-
Tibial (R)	Popliteal	11.3	8.8 mV	51.4
Sensory Nerve Conduction Study				
Median (L)	Palmar	1.6	247.0 µV	50.0
Ulnar (L)	F5	1.7	8.7 µV	46.0
Sural (R)	Mid-Calf	3.8	31.3 µV	34.4
F-Wave Study				
Median (L)	Wrist	F-lat: 25.3	Occurrence: 7/11 (64%)	-
Ulnar (L)	Wrist	F-lat: 25.7	Occurrence: 10/16 (63%)	-

**Figure 1:**
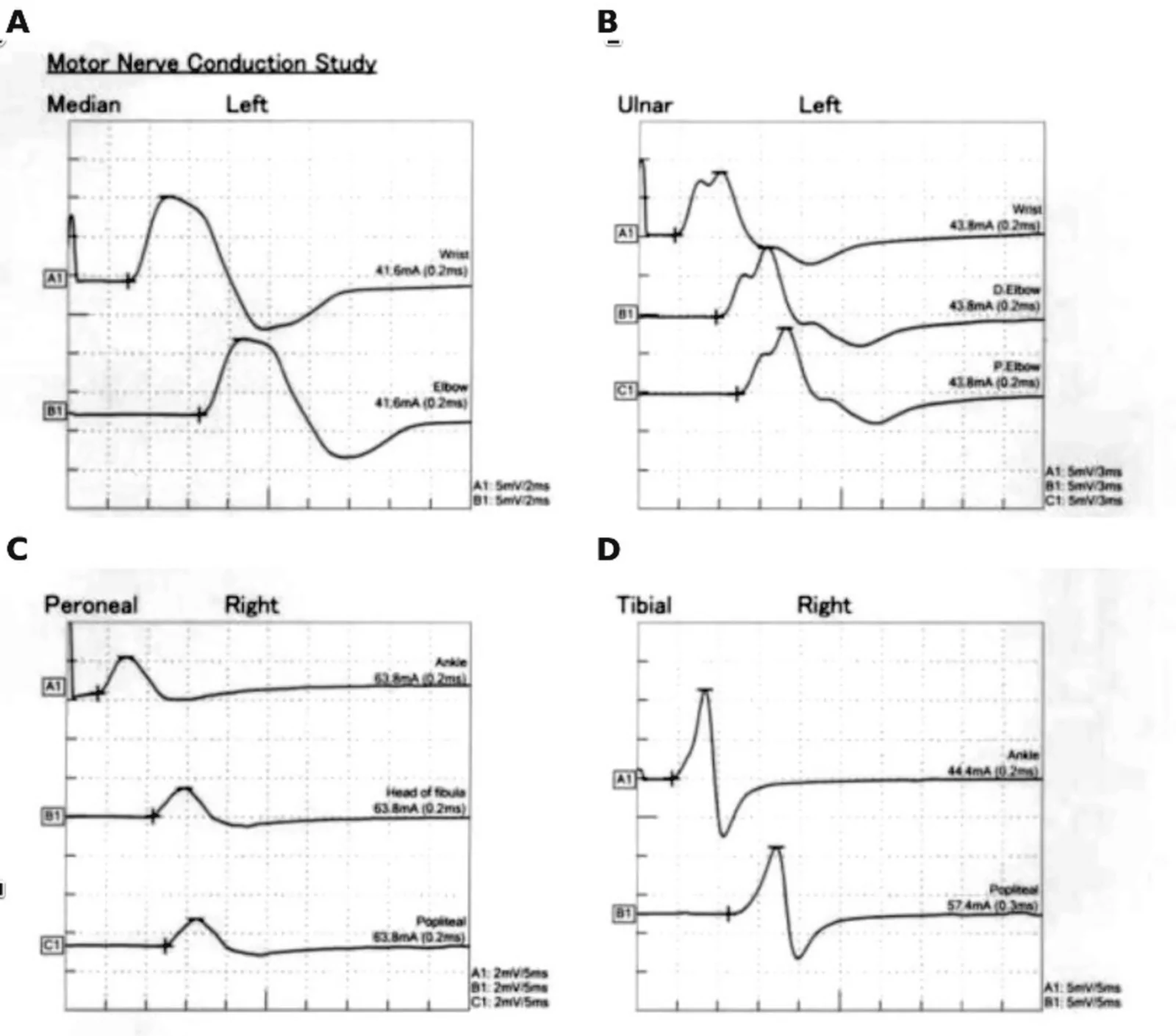
Representative motor nerve conduction study tracings, all within normal limits (A) Left median motor nerve, recorded at the wrist (A1) and elbow (B1). (B) Left ulnar motor nerve, recorded at the wrist (A1), distal elbow (B1), and proximal elbow (C1). (C) Right peroneal motor nerve, recorded at the ankle (A1), head of fibula (B1), and popliteal fossa (C1). (D) Right tibial motor nerve, recorded at the ankle (A1) and popliteal fossa (B1). In each panel, the y-axis represents the amplitude of the evoked compound muscle action potential, with vertical gain shown in millivolts (mV) per division for each trace; the x-axis represents time following stimulation, with sweep speed shown in milliseconds (ms) per division. Values labeled adjacent to each trace indicate the stimulus intensity and pulse duration (mA, ms) used to elicit the response.

**Table 2:** Needle electromyography findings MUAP: motor unit action potential

Muscle	Side	Insertional Activity	Spont. Activity	MUAP Duration/Amplitude	Recruitment	Interference Pattern
Iliopsoas	Left	Increased	None	Short duration, low amplitude	Full	Full
Vastus Lateralis	Left	Increased	None	Short duration, low amplitude	Full	Full
Tibialis Anterior	Left	Normal	None	Short duration, low amplitude	Full	Full

Electrocardiography showed normal sinus rhythm, and transthoracic echocardiography demonstrated preserved systolic function with an ejection fraction of 60%, with no history of aspiration with solids or liquids. Molecular testing confirmed a pathogenic homozygous c.464A>G (p.Asn155Ser) (NM_024854.4) missense variant in the PYROXD1 gene in the index patient. Targeted segregation testing subsequently confirmed the same homozygous variant in her affected brother, inherited from both parents, who were each clinically unaffected heterozygous carriers, consistent with autosomal recessive inheritance. Heterozygous carrier status for the variant was also identified in three of the index patient’s children (one daughter and two sons), establishing a fully characterized familial segregation pattern. This recurrent missense variant has been previously reported in individuals with PYROXD1-related myopathy and is supported by several lines of evidence, including occurrence in affected individuals, rarity in population databases, deleterious in silico predictions, and pathogenic ClinVar submissions. Taken together, these findings support classification as likely pathogenic or pathogenic under American College of Medical Genetics and Genomics/Association for Molecular Pathology criteria [7]; formal variant classification integrated segregation data, population frequency, computational predictions, and the reported cases discussed above. This genetic and segregation analysis confirmed the diagnosis of PYROXD1-related myopathy, establishing these siblings as the first molecularly confirmed family with this condition in North Africa.

As no disease-modifying therapy currently exists for PYROXD1-related myopathy, management was supportive. The patient was referred for physical therapy focused on gait training, stretching, and maintenance of proximal muscle strength, as well as for speech and respiratory therapy given her bulbar and respiratory symptoms. Weight loss and improved sleep hygiene resulted in resolution of her obstructive sleep apnea, while speech therapy provided instruction in compensatory swallowing techniques. The patient was scheduled for periodic clinical follow-up to monitor functional status and disease progression over time.

## Discussion

This case highlights a critical shift in our understanding of PYROXD1-related muscle disorders. While initial cohorts characterized this gene as a cause of severe, infantile-onset congenital myopathies, our family demonstrates that biallelic variants can manifest as a slowly progressive, adult-onset LGMD mimic with later respiratory and bulbar involvement.

The c.464A>G (p.Asn155Ser) variant identified in our patient alters a highly conserved amino acid residue within the pyridine nucleotide-disulfide oxidoreductase domain. Given the established role of this domain in PYROXD1 protein function [1,2], this substitution may plausibly affect protein localization and cellular oxidative stress responses; however, this proposed effect was not functionally assessed in our family. Notably, the same variant has been reported in geographically diverse cohorts, with variable clinical severity [1-4]. Such phenotypic variability may reflect the influence of modifier genes, epigenetic factors, environmental exposures, or other genetic background effects, although these possibilities remain hypothetical and were not directly assessed in our family.

Our family is only the second to be reported on the African continent, and, together with a recently reported unrelated Tunisian case harboring the same variant [6], underscores a significant gap in the literature regarding the true global prevalence and ethnic distribution of PYROXD1 mutations. Prior reported cases have predominantly emerged from European, North American, and Asian cohorts [1-5]. The identification of this family in Tunisia indicates that PYROXD1 should be considered in the differential diagnosis of unexplained or atypical LGMD phenotypes within North African populations.

A major diagnostic challenge in this case was the entirely normal serum CK level (113 IU/mL). In many classic muscular dystrophies, such as dystrophinopathies or sarcoglycanopathies, CK levels are markedly elevated; however, normal or only mildly elevated CK levels are frequently observed in congenital and myofibrillar myopathies, and this can mislead clinicians into prematurely excluding a primary muscle disease in adults [1,8]. Clinicians should therefore recognize that a normal CK level, particularly when accompanied by subtle skeletal or structural features, does not exclude an underlying myopathy. These findings are not specific to PYROXD1-related disease and are, in fact, more commonly associated with far more prevalent muscular dystrophies and congenital myopathies. Consequently, when targeted next-generation sequencing for these more common limb-girdle and congenital myopathy etiologies is negative, clinicians should broaden testing to a comprehensive myopathy panel capable of detecting rarer causes. When an adult patient presents with an apparent LGMD phenotype, characterized by normal or near-normal CK levels, coupled with scoliosis or hypermobility, this constellation should raise suspicion for an underlying genetic myopathy, with PYROXD1 considered among the differential diagnoses. In our patient, the presence of scoliosis and a high-arched palate contributed to this broader diagnostic suspicion.

This report has several limitations. Standardized quantitative functional measures (e.g., MRC sum score, Gowers’ sign, timed function tests, six-minute walk test) were not formally administered, and longitudinal follow-up data are not yet available to objectively confirm the rate of disease progression; both are recommended for future evaluation of this family. Muscle biopsy and muscle MRI were not performed, as a genetic-first diagnostic strategy was pursued once the familial pattern and phenotype were recognized, and functional validation of the variant’s effect on protein localization and oxidative stress handling was not undertaken in this family.

## Conclusions

Biallelic PYROXD1 mutations represent a rare but important etiology of adult-onset limb-girdle-like phenotypes, which may also include later respiratory and bulbar involvement. This case expands the known epidemiological footprint of the disease into Tunisia and North Africa.

Recognizing extra-muscular features such as scoliosis, a high-arched palate, joint hypermobility, and a nasal voice profile, although not specific to any single disorder, can prompt consideration of an underlying genetic myopathy, including PYROXD1-related disease. Increasing awareness of these phenotypes will allow clinicians to select appropriate broad-spectrum genetic testing panels early on, avoiding lengthy diagnostic odysseys for affected patients.
